# Safety, Tolerability and Pharmacokinetics of the eNAMPT-Neutralizing ALT-100 Mab in Healthy Volunteers

**Published:** 2024-09-18

**Authors:** Stan Miele, Thomas Polasek, Susanna Mantovani, Sara M. Camp, Joe G. N. Garcia

**Affiliations:** 1 Aqualung Therapeutics Corporation, 120 Scripps Way, Jupiter, FL 33458, United States.; 2 CMAX Clinical Research, Adelaide, Australia.; 3 Beyond Drug Development, Brisbane, Australia.

**Keywords:** eNAMPT, TLR4, DAMP, PD, PK, C_max_, SAEs

## Abstract

**Introduction:**

Human and preclinical studies have highlighted eNAMPT (extracellular nicotinamide phosphoribosyl transferase) as a druggable TLR4 ligand and DAMP involved in the pathobiology of diverse inflammatory, fibrotic and cancer disorders. This Phase 1 study assesses the safety, pharmacokinetics (PK) and pharmacodynamics (PD) of the humanized eNAMPT-neutralizing ALT-100 mAb as a strategy to address the unmet need for effective anti-inflammatory, anti-fibrotic therapeutics.

**Materials and Methods:**

Healthy male and female volunteers received a single intravenous ALT-100 dose (0.1, 0.4, 1.0, 4.0 mg/kg, n=24 dosed) or placebo (n=12 dosed) with 120-day monitoring (injection site, vital signs, hematology, coagulation, blood chemistry, urinalysis, PK/PD parameters, plasma biomarkers, anti-drug antibodies).

**Results:**

ALT-100 was well tolerated at all doses without clinically significant changes in local tolerability, vital signs, or laboratory safety parameters. Treatment-emergent adverse events (TEAEs) were unrelated to ALT-100 mAb dose (mild/moderate in severity), and AEs were transient and resolved without clinical sequelae. There were no serious adverse events (SAEs). Median ALT-100 mAb plasma levels peaked at 0.62 hour after dosing (all doses). The mean maximum mAb plasma concentration (C_max_) and mAb elimination half-life (T1/2) all increased in a dose-related manner between 0.4 mg/kg (17 days) and 4 mg/kg (27 days).

**Conclusions:**

Single intravenous ALT-100 mAb doses are well tolerated in healthy participants with dose proportional PK and elimination half-life.

## Introduction

Damage-associated molecular pattern molecules (DAMPs) are actively secreted or passively released by damaged cells as an innate immunity mechanism to amplify inflammatory cascades via ligation of pattern recognition receptors (PRRs) including Toll-Like Receptors (TLRs). For example, high-mobility box group 1 (HMGB1) is a dually functioning cytozyme implicated in multiple inflammatory disorders [1] that intracellularly functions as a nonhistone nucleoprotein. However, when secreted or released from dying cells, HMGB1 exhibits strong DAMP properties via binding to TLR2, TLR4 and RAGE [2,3]. Similarly, our prior genomic–intensive approaches identified a novel cytozyme, eNAMPT (extracellular nicotinamide phosphoribosyl-transferase) as a highly evolutionarily-conserved DAMP and master regulator of inflammatory cascades via ligation of TLR4. Intracellular NAMPT is a major NAD-synthesizing enzyme with anti-apoptotic properties via SIRT1 [4]. However, in response to multiple cellular stressors (viral or bacterial infection, mechanical stress, hypoxia), eNAMPT is released into the circulation with plasma eNAMPT levels linked to disease severity/mortality in severe acute inflammatory lung disorders (including ARDS and sepsis) [5–7] and to chronic autoimmune disorders such as inflammatory bowel disease [8], systemic lupus erythematosus [9] and nonalcoholic steatohepatitis [10] as well as pulmonary hypertension [11] and prostate cancer [12,13].

To address the unmet need for therapies that reduce the severity of acute and chronic inflammatory disorders, we created multiple murine eNAMPT-neutralizing mAbs and after extensive subcloning, selected two eNAMPT-neutralizing murine mAbs. Each mAb underwent humanization with the 50 humanized variants generated (Fusion Antibody Inc, Belfast UK) undergoing screening for potency in attenuating eNAMPT-induced inflammatory lung injury. The ALT-100 mAb was selected as lead mAb and underwent optimization of complementarity-determining regions (CDR) sequences to reduce immunogenicity. Following successful ALT-100 mAb stable cell line development and conversion of the research cell bank (RCB) to a Master Cell Bank (MCB) (CMAB/Wuxi Biologics, Shanghai, China), a 200L GMP Bioreactor run was completed with mAb expression at ~6 gm/L yielding sufficient GMP ALT-100 material for multiple trials including the current Phase 1A study.

## Materials and Methods

### Study Design

This was a first-in-human (FIH), double-blind, randomized, placebo-controlled single ascending dose (SAD) study investigating the safety, tolerability and pharmacokinetic (PK) profile of intravenously delivered ALT-100 mAb at doses of 0.1 mg/kg, 0.4 mg/kg, 1.0 mg/kg and 4 mg/kg, to healthy participants. A total of 32 participants were enrolled, randomized and received a single IV infusion of ALT-100 or placebo. Within each of the 4 cohorts, 6 participants were dosed with ALT-100 and 2 participants were dosed with placebo. Two sentinel participants were initially dosed with ALT-100 or placebo in each cohort prior to the rest of the cohort being dosed.

The study comprised a screening period of up to 28 days prior to enrollment, the day of treatment on Day 1 and a follow up period to Day 120. Participants remained in the clinical trial unit (CRU) from day −1 to Day 3 and returned to the CRU on an outpatient basis on Day 8 (±1 day), Day 15 (±1 day), Day 22 (±2 days), Day 29 (±3 days), Day 60 (±7 days), Day 90 (±7 days), and Day 120 (±7 days), for safety and other assessments.

For dose finding in a FIH study, this trial followed the recommendations of the United States (US) Food and Drug Administration (FDA) (Guidance for Industry: Estimating the Maximum Safe Starting Dose in Initial Clinical Trials for Therapeutics in Adult Healthy Volunteers. U.S. Department of Health and Human Services Food and Drug Administration Center for Drug Evaluation and Research [CDER] July 2005). The safe starting dose was based on toxicity studies in animals. In a 14-day toxicity study in rats and minipigs where 2 doses of ALT-100 were administered by IV injection, and given 1 week apart, the no-observed-adverse-effect-level (NOAEL) was determined to be 50 mg/kg/day in both species. As per FDA Guidance 2005 (Estimating the maximum safe starting dose in initial clinical trials for therapeutics in adult healthy volunteers), a large molecular weight protein (>100kDa) administered intravascularly should be dose normalized relative to body weight i.e., mg/kg. ALT-100 is a large molecular weight mAb (147 kDa), and therefore safety margins based on the animal NOAELs relative to the human starting dose were estimated based on a mg/kg basis. On a mg/kg basis the clinical starting dose of 0.1 mg/kg was 500 times lower than the NOAELs in rat and minipig. On the basis of the total mg dose administered to the rat, the NOAEL was 3 times the clinical start dose (6 mg, assuming a body weight of 60kg), owing to the small body weight of the animals, whereas the safety margin estimate based on the mg dose given to the minipig was 83 times the clinical starting dose (6 mg, assuming a 60 kg body weight).

### Study Subjects

The inclusion criteria required healthy male or female volunteers between 18 to 55 years of age (inclusive) with the ability to understand the nature of the study and any risks involved in participation. Volunteers needed to be willing to cooperate and comply with the protocol restrictions and requirements and be capable and willing to provide written informed consent. Females of childbearing potential (WOCBP) were required to have a negative pregnancy test at screening and admission. WOCBP and male participants who were not sterile or abstinent and were engaged in sexual relations with a WOCBP were required to use highly effective contraception from screening until study completion. Eligible participants exhibited a mean body weight of greater than or equal to 50 kg and a body mass index of (BMI) of 18 to 32 kg/m2 at screening. A summary of the demographics for the study population are in Table 1.

### Ethics

The study was performed at a single center in Australia and only commenced after independent review and approval by a human research ethics committee. Informed written consent was obtained from all participants before any procedures were performed. The study was conducted according to the protocol and the ethical principles that have their origins in the Declaration of Helsinki including the ICH Good Clinical Practice (GCP) Consolidated Guideline E6 (R2), annotated with comments from the Australian Therapeutics Good Administration, 2018, and all applicable national and local laws and regulations. The study was registered on clinicaltrials.gov under the identifier NCT05426746. The study data was independently monitored by a company independent of the clinical trial site or Aqualung Therapeutics, who were appointed to verify that the study was conducted in accordance with current GCP, regulatory requirements, the protocol and that the data were authentic, accurate and complete.

### Study Procedures

#### Safety Measurements:

Safety of participants were assessed by physical examination, vital signs including respiratory rate, heart rate, systolic and diastolic blood pressure and body temperature, a 12-lead electrocardiogram (ECG), laboratory blood tests including hematology, clinical chemistry and coagulation, and urinalysis. Adverse events (AE) were reported for all participants from the time the participant informed consent form was completed and until the end of study visit on Day 120 ± 7 days.

#### Anti-drug Antibody Monitoring:

Blood samples were collected for ADA monitoring in serum collected prior to study treatment, and on Day 8, 15, 29, 60, 90 and 120 post dosing (Agilex Biolabs, Australia). Additional samples were collected if any participant exhibited signs or symptoms of infusion-related reaction. Samples were analyzed using a validated immunoassay.

#### Pharmacokinetic (PK) Concentrations:

Blood samples for the determination of plasma PK of ALT-100 were obtained from participants. Blood samples were collected prior to dosing (within 1 hour), at 10, 20, 30, and 45 minutes, 1, 2, 4, 8, 24, and 48 hours after the end of dosing, as well as on Day 8, 15, 22, 29, 60, 90 and 120. The actual sample collection dates and times were reported on the ‘Pharmacokinetics Blood Sample Collection’ eCRF. For plasma samples, the actual elapsed time (hours) since start of investigational product infusion was calculated as the difference between the date time of the sample collection at the nominal time point and the date time of the start of the IP infusion. The time deviation at each time point (hours), defined as the difference between the nominal (planned) and actual collection times was calculated as the difference between the nominal collection time point value (i.e., 2 hours post infusion will be 2 hours) and the elapsed time based on the actual collection date and time.

#### Plasma Measurements of Human Cytokines:

Blood samples for biomarkers and cellular parameters (neutrophil, monocyte and lymphocyte counts) were collected prior to study treatment and post treatment on Day 1 (6 hrs.), Day 2, 3, 8, 15, and 29. Human plasma samples were analyzed for levels of eNAMPT, IL-6, IL-8, TNF and IL-1RA (Agilex Biolabs, Australia).

## Results

### Demographics:

The study was conducted between 30 June 2022 and 18 May 2023. A summary of the demographics for the study population are in Table 1. Thirty-two participants met the eligibility criteria and were enrolled, randomized and received a single IV infusion of ALT-100 or placebo. Overall, 21 of 32 (65.6%) participants enrolled were female. The mean age of all participants was 32 years (range 18 to 53 years). All female participants were of childbearing potential except for one female in cohort 1 (dosed with placebo). Participants had a mean body weight of 73.9 kg (range 52.7 to 106.8 kg) and a mean BMI of 25.4 kg/m2 (19–31.3 kg/m2 range) at screening. No female participant was pregnant and all participants agreed to adhere to the contraception requirements defined in the study protocol. The majority of participants were White (28 of 32 [87.5%]), with 2 Asian, 1 Latino and 1 half Asian, half Caucasian.

**Figure 1:**
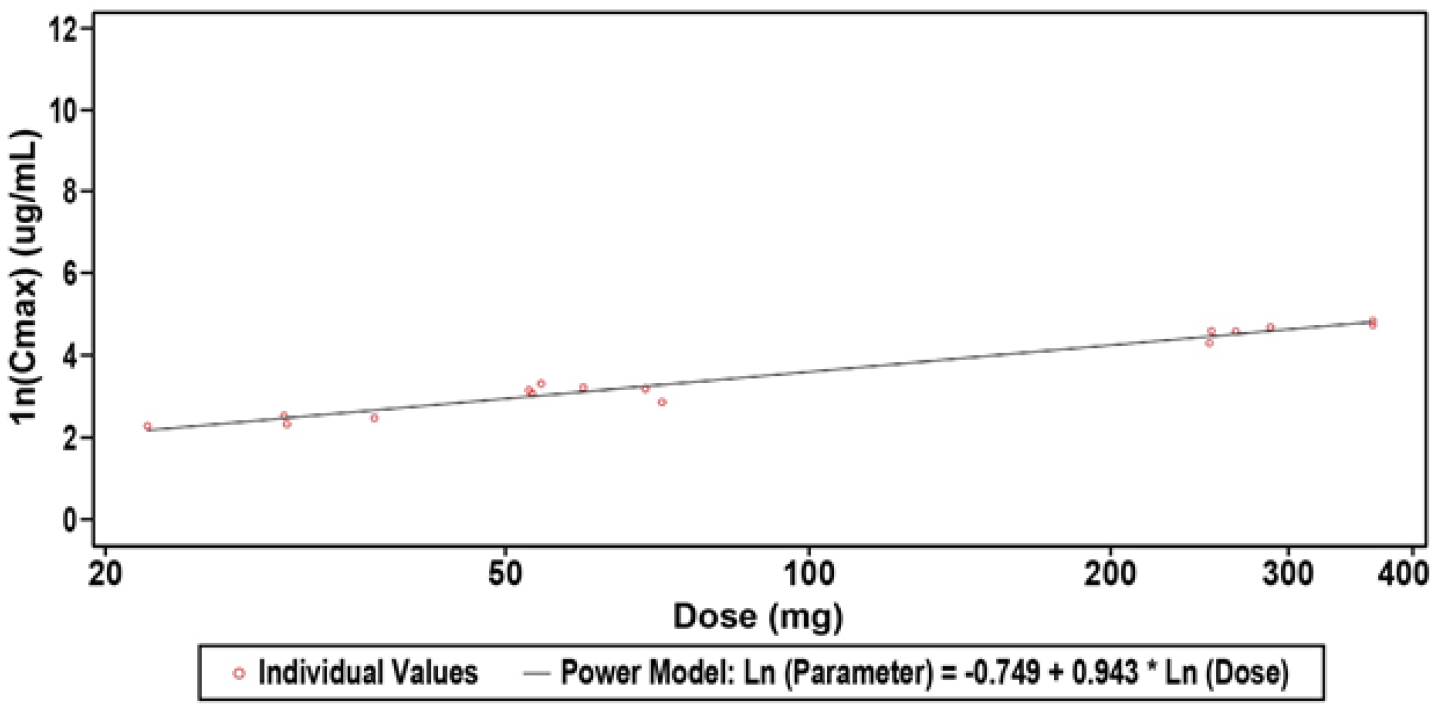
Scatterplots of Natural Log-Transformed Plasma ALT-100 Pharmacokinetic Parameters for Dose Range 0.4 to 4.0 mg/kg ALT-100 (PK Population). Shown is the clear dose response observed with the higher dose of ALT-100mAb with the standard low dose having a t1/2 of ~16 days and the moderate dose having a t1/2 of ~22 days. **Abbreviations:** SD: Standard Deviation. This was a sensitivity analysis for the 0.4 to 4 mg/kg dose range. A power model was used to assess dose proportionality: Natural log-transformed (PK Parameter Result) = ß0 + ß1* Natural Log-transformed (Dose).

Full compliance with study drug administration was observed in all participants, and all received the complete IV dose of planned ALT-100 or placebo treatment, on Day 1. The number of participants screened, randomized, enrolled and completing the study is outlined in Figure 1 along with a summary of analysis populations. Two participants, 1 in the ALT-100 1 mg/kg group and 1 in the placebo group, discontinued early due to withdrawal of their consent. The participant in Cohort 2 (placebo treatment) completed the study to Day 29 and the participant in Cohort 3 (ALT-100, 1 mg/kg) completed the study to Day 85.

### Safety and Tolerability

Overall, 54 TEAEs were reported in 22 of 32 (68.8%) participants in the study. Of these TEAEs, 15 events reported in 9 of 32 (28.1%) participants were deemed related to the study drug (Table 2). None of the participants had AEs leading to death, nor were any SAEs reported during this study. None of the AEs reported in the study were Grade 3 (severe). Grade 2 (moderate) events were reported in 5 instances by 4 participants. None of them were in the placebo group. None of the Grade 2 (moderate) events were reported in any of the sentinel participants, and no stopping criteria were met during the study. All other AEs reported in the study were Grade 1 (mild) in severity. No dose limiting toxicity, nor infusion-related reactions (IRR) were recorded for any of the participants in the study. All AEs reported in the study resolved/recovered, and none were ongoing at the completion of the study. No participant in the study reported any AEs leading to study discontinuation or study drug withdrawal during the conduct of this study. A moderate event of anxiety (shortness of breath) on Day 1, was reported in a participant in the 4.0 mg/kg ALT-100 treatment group (Cohort 4) and led to an interruption in study drug administration. The event, which was deemed possibly related to the study drug, occurred 5 minutes into the start of study drug infusion, and resolved within 1 minute, without any intervention. Dosing of the study drug resumed afterwards, and the participant received the complete dose of ALT-100.

The most commonly reported TEAEs by system organ class (SOC) were general disorders and administration site conditions, reported by at least 1 participant in each treatment group, and overall reported by 11 of 32 (34.4%) participants in the study, with a total of 13 events. These included: fatigue, pyrexia, feeling hot, vessel puncture site bruise, catheter site pain, catheter site related reaction, and injection site erythema.

The second most commonly reported TEAEs by SOC were nervous system disorders, reported by at least 1 participant in each treatment group with the exception of participants treated with the highest dose of ALT-100 (4.0 mg/kg, Cohort 4), who did not report any TEAEs for this SOC. Overall, 9 of 32 (28.1%) of participants reported at least 1 nervous system disorder, with a total of 10 events, mostly headache (8 events) being reported. Headache was also the most frequently reported TEAE related to study drug administration, with a total of 4 events reported in 4 of 24 (16.7%) participants in the pooled ALT-100 treated group (Group 2). None of the placebo participants had any event of headache that was deemed study drug related. All other TEAEs that were reported as study drug related occurred in no more than 1 participant each, in no more than 1 instance and included: somnolence, fatigue, ECG PR prolongation, flushing, catheter site pain, tonsillitis, anxiety, insomnia, feeling hot, bronchospasm and hypersensitivity (allergic reaction, starting and resolving on Day 90) (see Table 2).

None of the investigated hematologic, coagulation, clinical chemistry or urinalysis parameters demonstrated any clinically significant change from baseline, including mean, median, minimum and maximum values for any of the treatment groups, and none met the criteria for a TEAE. Overall, there was no clinically-relevant pattern of hematologic, coagulation, clinical chemistry or urinalysis parameter changes evident with the administration of ALT-100 at any of the dose levels tested in the study compared to placebo, or between ALT-100 dose levels in these healthy adult participants at the time points assessed. None of the vital signs recorded at any of the timepoints in any of the treatment groups were deemed clinically significant by the Study Principal Investigator. Overall, there was no clinically-relevant pattern of vital signs parameters changes evident with the administration of ALT-100 at any of the dose levels tested in the study compared to placebo, or between ALT-100 dose levels in these healthy adult participants at the time points assessed. The 12-lead ECG results recorded between Day 1 (end of infusion) and Day 8 for 1 participant in the 0.4 mg/kg ALT-100 treatment group (Cohort 2) were deemed clinically significant by the Investigator, and resulted in the TEAE of mild ECG PR prolongation deemed possibly related to study drug. None of the other 12-lead ECG results met the criteria for a TEAE.

### Immunogenicity and Anti-Drug Antibody (ADA)

None of the participants in the placebo group exhibited the presence of anti-ALT-100 antibodies at any of the time points analyzed. Sporadic positive ADA test results were reported in a total of 5 participants treated with ALT-100. In each of the active treatment groups, no more than 2 participants had detectable levels of ADAs, and at no more than 3 timepoints each. Two participants in the pooled ALT-100 group had an ADA titer that was detected at baseline (before drug administration), 1 participant in the 1.0 mg/kg ALT-100 (Cohort 3), and 1 participant in the 4 mg/kg ALT-100 (Cohort 4) treatment group, where the titer was borderline positive. Overall, the incidence of ADAs in participants’ serum was low and transient. A limitation of the analysis is posed by the fact that ALT-100 was administered as a single (not multiple) dose to participants in this study. No IRR were reported in the study, and no additional samples (other than those scheduled) for ADA determination were collected from participants in the study.

### Exploratory Biomarkers

Overall, no clinically-significant differences from baseline were identified in the count of neutrophils, neutrophils/leukocytes, monocytes, monocytes/leukocytes, lymphocytes, or lymphocytes/ leukocytes in whole blood samples among ALT-100 dose treatment groups, or between pooled ALT-100 treated participants and pooled placebo participants at 4 and 6 hours post dose, on Day 2, 3, 8, 15, 22 and 29 visits. No clinically-relevant observations were made regarding the plasma concentration of the reported biomarkers (eNAMPT, IL-1 RA, IL-6, IL-8, TNF). The results obtained for the analyzed PD parameters were not unexpected, given that the study population was composed of healthy adult subjects.

### Pharmacokinetics (PK)

**Figure 2:**
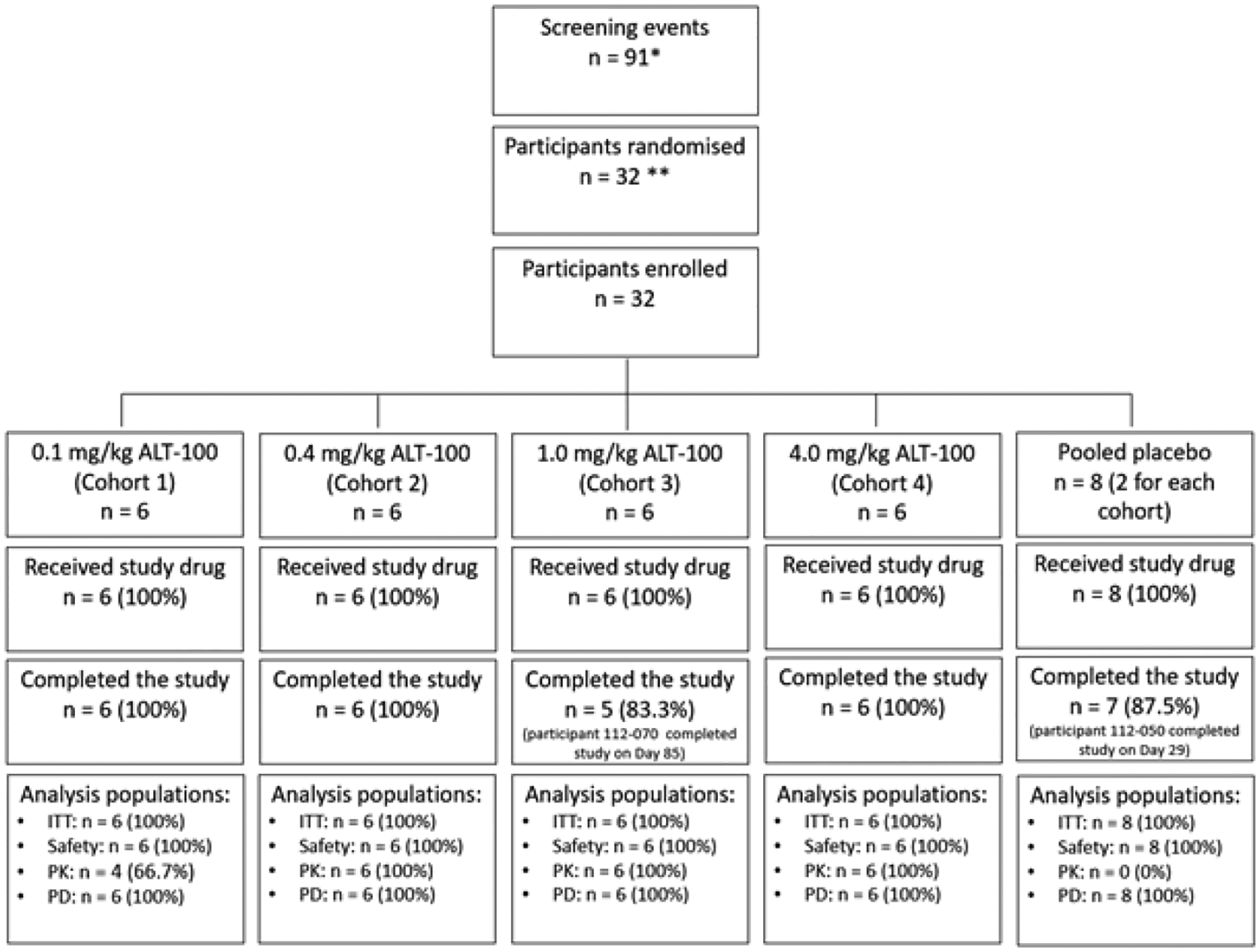
Study Diagram. Each cohort had two placebo participants and in the ‘intent to treat’ population there was a very high completion rate in three of the 4 cohorts. One patient in cohort 3 was unable to complete the day 120 follow up visit. **Abbreviations:** ITT, intent to treat; n, number; PD, pharmacodynamics, PK, pharmacokinetics; *, this number includes participants who were rescreened and allocated a new screening number, the total number of screen failures was 37 and 11 participants withdrew their consent ahead of enrollment. **, in 2 cases a randomization number was reallocated to allow pharmacy to make drug, prior to the participant being officially randomized. Two participants failed eligibility criteria and were not dosed. Two new participants were randomized and dosed correctly.

The PK of ALT-100 administered by IV infusion at doses of 0.1, 0.4, 1.0 and 4.0 mg/kg is reported in Table 3. The observed C_max_ for ALT-100 appeared to be close to dose proportional for all dose levels tested while AUC0-t demonstrated above dose proportional dependence for the complete dose range of 0.1 to 4.0 mg/kg ALT-100. The accuracy of the dose proportionality analysis was affected by the small number of quantifiable concentrations for the 0.1 mg/kg ALT-100 dose level. The descriptive evaluation of dose normalized PK parameters for the range 0.4 to 4.0 mg/kg indicated potential dose proportionality for this range (Table 4, Figure 2). This analysis confirmed that both C_max_ and AUC0-t met all the criteria for dose proportionality within this range. AUC0-inf observations were limited. The mean terminal elimination half-life (t1/2) of ALT-100, was determined to be dose dependent and ranged approximately from 17 to 27 days for dose levels 0.4 to 4.0 mg/kg ALT-100. Clearance (CL) was in line with the t1/2. Terminal volume of distribution (Vz) was 4.8 L for 1 of 6 participants in 1.0 mg/kg ALT-100 dose group and 8.1 L for 5 of 6 participants in the 4.0 mg/kg ALT-100 dose group. The variability of Vz at 4.0 mg/kg ALT-100 dose level was 24.7%.

## Discussion

Following the strong efficacy demonstration of the ALT-100 mAb in preclinical models of acute [14–16] and chronic disease [9,10,12,17–20], we now report results of a FIH, Phase 1, randomized, double-blind, placebo-controlled study conducted at a single center in Australia to investigate the safety, tolerability, and PK profile of single ascending doses of IV infused ALT-100 administered in healthy participants. The PD effects and immunogenicity of ALT-100 were also explored. This manuscript presents final analysis findings from this Phase 1, clinical trial on the safety and PK of an investigational humanized mAb against extracellular eNAMPT, a novel and potent TLR4 activating DAMP ligand in healthy adults ≥18 years. A total of 32 participants met the eligibility criteria, and were enrolled, randomized, and received a single IV infusion of ALT-100 or placebo on Day 1. Participants were randomized at a 3:1 ratio (active: placebo) to 1 of 4 planned SAD cohorts. In the study there were 6 participants who received ALT-100 at 0.1 mg/kg (Cohort 1), 6 participants who received ALT-100 at 0.4 mg/kg (Cohort 2), 6 participants who received ALT-100 at 1.0 mg/kg (Cohort 3), 6 participants who received ALT-100 at 4.0 mg/kg (Cohort 4), and 8 participants who received placebo treatment (2 placebo treated participants in each cohort). Overall, 23 of 32 (71.9%) participants had at least 1 protocol deviation, and these occurred in all cohorts and treatment groups. The majority of the protocol deviations reported in the study were classified as minor, and there were no major deviations that met the definition of a serious breach. Minor protocol deviations were mainly due to missed procedures/assessments and procedures/assessments conducted outside of the protocol window. Major protocol deviations occurred in 3 of 32 (9.4%) participants overall, and were all due to missed procedures and/or assessments or missed visits. COVID-19 infection occurred in 2 instances in 2 participants in the study. None of these 2 events led to any protocol deviation, and COVID −19 did not have any impact on the study conduct. Demographic characteristics of age, race and ethnicity, height, weight and BMI at baseline were broadly consistent across all treatment groups. Demographic distribution of sex was also broadly consistent across all treatment groups, apart for the ALT-100 1.0 mg/kg treatment group in Cohort 3, who were all female.

Two participants, 1 in the ALT-100 group dosed with 1 mg/kg (Cohort 3) and 1 in the pooled placebo group, discontinued the study earlier than planned due to withdrawal of their consent, on Days 85 and 29 respectively. All remaining participants who were assigned to treatment and dosed completed the study as planned.

Overall ALT-100 was well tolerated and safe at the doses tested in healthy participants. The results from this Phase 1 FIH study support the further investigation of ALT-100 delivered by the IV route in patients. The PK analysis for ALT-100 indicates is consistent with the characteristics of a mAb and dose proportionality was seen with the dose range of 0.4 to 4 mg/kg.

Thus, in summary, a new chemical entity that targets eNAMPT must undergo rigorous preclinical, IND-enabling animal studies before entering human clinical trials. ALT-100 mAb has high affinity to eNAMPT and this P1A study in healthy human volunteers demonstrated the drug’s overall safety, which mirrored the safety which was demonstrated in all the IND-enabling studies in rats and minipigs. The overall safety results certainly underscore the importance of advancing this novel compound into ongoing clinical trials in a disease state population such as acute respiratory distress syndrome (ARDS). Aqualung Therapeutics, with clearance from the FDA to proceed, has initiated the PUERTA study (NCT05938036), a Phase 2A clinical trial currently enrolling subjects who are diagnosed with moderate/severe ARDS.

## Conclusions

ALT-100 mAb was well tolerated and safe at all doses tested (0.1, 0.4, 1.0, and 4.0 mg/kg) in healthy adult volunteers. The results from this Phase 1 FIH study support the further investigation of ALT-100 delivered IV for the treatment of patients with ARDS. The PK analysis indicated that the PK characteristics of ALT-100 are consistent with the characteristics of monoclonal antibodies (mAbs), and dose proportionality of ALT-100 within the range of dose levels from 0.4 to 4.0 mg/kg was proven.

## Figures and Tables

**Table 1:** Summary of Demographic Information (n=32).

	Placebo (n=8)	ALT-100 Treatment (n=24)
Age (years)	32.1	32.0
Sex (female) n(%)	4 (50)	17 (70.8)
Height (cm)	173.1	168.6
Weight (kg)	78.68	72.33
BMI (kg/m^2^)	25.83	25.26

Summary of demographics and baseline characteristics of the intention-to-treat population. The data are mean values and in the case of sex is number (%).

**Table 2:** Incidence of Treatment-Emergent Adverse Events by System Organ Class and Preferred Term (Safety Population).

System Organ Class (Soc) Preferred Term (Pt)	0.1 mg/kg ALT-100 (N=6) n (%) E	0.4 mg/kg ALT-100 (N=6) n (%) E	1.0 mg/kg ALT-100 (N=6) n (%) E	4.0 mg/kg ALT-100 (N=6) n (%) E	Pooled ALT-100 (N=24) n (%) E	Pooled Placebo (N=8) n (%) E	All Participants (N=32) n (%) E
At Least One TEAE	6 (100) 15	4 (66.7) 12	5 (83.3) 11	3 (50.0) 7	18 (75.0) 45	4 (50.0) 9	22 (68.8) 54

**General Disorders and Administration Site Conditions**	4 (66.7) 5	2 (33.3) 3	2 (33.3) 2	1 (16.7) 1	9 (37.5) 11	2 (25.0) 2	11 (34.4) 13
Vessel Puncture Site Bruise	2 (33.3) 2	1 (16.7) 1	1 (16.7) 1	0	4 (16.7) 4	1 (12.5) 1	5 (15.6) 5
Fatigue	1 (16.7) 1	1 (16.7) 1	0	0	2 (8.3) 2	0	2 (6.3) 2
Pyrexia	1 (16.7) 1	1 (16.7) 1	0	0	2 (8.3) 2	0	2 (6.3) 2
Catheter Site Pain	0	0	0	0	0	1 (12.5)1	1 (3.1) 1
Catheter Site Related Reaction	1 (16.7) 1	0	0	0	1 (4.2) 1	0	1 (3.1) 1
Feeling Hot	0	0	0	1 (16.7) 1	1 (4.2) 1	0	1 (3.1) 1
Injection Site Erythema	0	0	1 (16.7) 1	0	1 (4.2) 1	0	1 (3.1) 1

**Nervous System Disorders**	3 (50.0) 4	2 (33.3) 2	2 (33.3) 2	0	7 (29.2) 8	2 (25.0) 2	9 (28.1) 10
Headache	2 (33.3) 3	1 (16.7) 1	2 (33.3) 2	0	5 (20.8) 6	2 (25.0) 2	7 (21.9) 8
Somnolence	1 (16.7) 1	0	0	0	1 (4.2) 1	0	1 (3.1) 1
Syncope	0	1 (16.7) 1	0	0	1 (4.2) 1	0	1 (3.1) 1

**Gastrointestinal Disorders**	1 (16.7) 1	1 (16.7) 2	0	0	2 (8.3) 3	2 (25.0) 3	4 (12.5) 6
Nausea	0	0	0	0	0	2 (25.0) 2	2 (6.3) 2
Abdominal Pain	0	1 (16.7) 1	0	0	1 (4.2) 1	0	1 (3.1) 1
Diarrhoea	0	1 (16.7) 1	0	0	1 (4.2) 1	0	1 (3.1) 1
Gastrooesophageal Reflux Disease	1 (16.7) 1	0	0	0	1 (4.2) 1	0	1 (3.1) 1
Vomiting	0	0	0	0	0	1 (12.5)1	1 (3.1) 1

**Respiratory, Thoracic and Mediastinal Disorders**	1 (16.7) 2	1 (16.7) 1	2 (33.3) 2	0	4 (16.7) 5	0	4 (12.5) 5
Bronchospasm	0	0	1 (16.7) 1	0	1 (4.2) 1	0	1 (3.1) 1
Cough	1 (16.7) 1	0	0	0	1 (4.2) 1	0	1 (3.1) 1
Oropharyngeal Pain	0	0	1 (16.7) 1	0	1 (4.2) 1	0	1 (3.1) 1
Sinus Congestion	1 (16.7) 1	0	0	0	1 (4.2) 1	0	1 (3.1) 1
Tonsillar Hypertrophy	0	1 (16.7) 1	0	0	1 (4.2) 1	0	1 (3.1) 1

**Infections And Infestations**	1 (16.7) 1	1 (16.7) 1	1 (16.7) 1	1 (16.7) 1	4 (16.7) 4	0	4 (12.5) 4
Covid-19	0	1 (16.7) 1	0	1 (16.7) 1	2 (8.3) 2	0	2 (6.3) 2
Pharyngitis	1 (16.7) 1	0	0	0	1 (4.2) 1	0	1 (3.1) 1
Tonsillitis	0	0	1 (16.7) 1	0	1 (4.2) 1	0	1 (3.1) 1

**Immune System Disorders**	1 (16.7) 1	1 (16.7) 1	0	1 (16.7) 1	3 (12.5) 3	0	3 (9.4) 3
Seasonal Allergy	1 (16.7) 1	1 (16.7) 1	0	0	2 (8.3) 2	0	2 (6.3) 2
Hypersensitivity	0	0	0	1 (16.7) 1	1 (4.2) 1	0	1 (3.1) 1

**Vascular Disorders**	0	0	1 (16.7) 2	1 (16.7) 1	2 (8.3) 3	0	2 (6.3) 3
Catheter Site Haematoma	0	0	1 (16.7) 1	0	1 (4.2) 1	0	1 (3.1) 1
Flushing	0	0	1 (16.7) 1	0	1 (4.2) 1	0	1 (3.1) 1
Thrombosis	0	0	0	1 (16.7) 1	1 (4.2) 1	0	1 (3.1) 1

**Injury, Poisoning and Procedural Complications**	0	0	1 (16.7) 1	1 (16.7) 1	2 (8.3) 2	0	2 (6.3) 2
Dermatitis Contact	0	0	0	1 (16.7) 1	1 (4.2) 1	0	1 (3.1) 1
Sunburn	0	0	1 (16.7) 1	0	1 (4.2) 1	0	1 (3.1) 1

**Musculoskeletal and Connective Tissue Disorders**	0	1 (16.7) 1	1 (16.7) 1	0	2 (8.3) 2	0	2 (6.3) 2
Myalgia	0	1 (16.7) 1	0	0	1 (4.2) 1	0	1 (3.1) 1
Pain In Extremity	0	0	1 (16.7) 1	0	1 (4.2) 1	0	1 (3.1) 1

**Psychiatric Disorders**	0	0	0	2 (33.3) 2	2 (8.3) 2	0	2 (6.3) 2
Anxiety	0	0	0	1 (16.7) 1	1 (4.2) 1	0	1 (3.1) 1
Insomnia	0	0	0	1 (16.7) 1	1 (4.2) 1	0	1 (3.1) 1

**Reproductive System and Breast Disorders**	0	0	0	0	0	2 (25.0) 2	2 (6.3) 2
Dysmenorrhoea	0	0	0	0	0	1 (12.5) 1	1 (3.1) 1
Vaginal Discharge	0	0	0	0	0	1 (12.5) 1	1 (3.1) 1

**Ear and Labyrinth Disorders**	1 (16.7) 1	0	0	0	1 (4.2) 1	0	1 (3.1) 1
Ear Pain	1 (16.7) 1	0	0	0	1 (4.2) 1	0	1 (3.1) 1

**Investigations**	0	1 (16.7) 1	0	0	1 (4.2) 1	0	1 (3.1) 1
Electrocardiogram PR Prolongation	0	1 (16.7) 1	0	0	1 (4.2) 1	0	1 (3.1) 1

Treatment-emergent adverse events (TEAEs) are defined as adverse events (AEs) which commence or worsened on or after the time of start of study drug administration. Participants who experienced multiple events within a category are counted only once in the specific category (n), however each instance of the event is counted (E). Percentage (%) are calculated based on the number of participants in the relevant population (N). Adverse event terms were coded using Medical Dictionary for Regulatory Activities (MedDRA) Version 25.0.

**Table 3:** Plasma ALT-100 Pharmacokinetics.

Dose of ALT-100 Parameter (unit)	N	Mean (SD)	CV (%)	Median	Minimum	Maximum	Geometric Mean	Geometric CV (%)
0.1 mg/kg (N=4)								
AUC_0-t_ (h*ug/mL)	4	2050.33 (4093.099)	199.6	3.84	3.6	8190.0	25.76	160027.6
C_max_ (ug/mL)	4	5.64 (5.8568)	103.9	2.725	2.67	14.42	4.11	100.7
T_max_ (h)	4	-		0.625	0.33	0.82	-	-
0.4 mg/kg (N=6)								
AUC_0-t_ (h*ug/mL)	4	2549.94 (522.690)	20.5	2453.40	2021.2	3271.7	2511.76	20.1
C_max_ (ug/mL)	4	11.013 (1.2930)	11.7	10.97	9.71	12.41	10.956	11.8
T_max_ (h)	4	-	-	0.625	0.33	0.77	-	-
t_½_ (h)	2	409.103 (7.4384)	1.8	409.10	403.84	414.36	-	-
1.0 mg/kg (N=6)								
AUC_o-inf_ (h*ug/mL)	1	10660.18 (−)	-	10660.18	10660.2	20660.2	10660.18	-
AUC_0-t_ (h*ug/mL)	6	7156.19 (1973.783)	27.6	7427.12	4593.2	9043.6	6916.13	29.8
C_max_ (ug/mL)	6	23.015 (3.3554)	14.6	23.600	17.37	27.27	22.796	15.5
T_max_ (h)	6	-	-	0.625	0.50	0.77	-	-
t_½_ (h)	3	504.397 (68.7401)	13.6	516.383	430.45	566.36	-	-
CL (L/h)	1	0.0065 (−)	-	0.0065	0.006	0.006	0.0065	-
V_z_ (L)	1	4.808 (−)	-	4.808	4.81	4.81	4.808	-
4.0 mg/kg (N=6)								
AUC_o-inf_ (h*ug/mL)	5	36421.27 (6910.076)	19.0	39283.10	26579.1	42499.3	35854.21	20.4
AUC_0-t_ (h*ug/mL)	6	30909.70 (7711.583)	24.9	32447.58	21291.1	39550.4	30055.80	26.9
C_max_ (ug/mL)	6	103.142 (17.8150)	17.3	103.32	73.93	126.11	101.76	18.5
T_max_ (h)	6	-	-	0.625	0.5	24.0	-	-
t_½_ (h)	5	651.935 (102.7980)	15.8	636.077	534.88	804.71	-	-
CL (L/h)	5	0.0088 (0.0029)	33.0	0.0079	0.006	0.014	0.0085	30.2
V_z_ (L)	5	8.072 (2.1515)	26.7	7.307	6.08	11.72	7.872	24.7

**Abbreviations:** AUC0-t, area under the concentration-time curve from time zero (Predose) to last quantifiable concentration at time t; AUC0-inf, area under the concentration-time curve from time zero (Predose) extrapolated to infinity; CL, total body clearance; C_max_, maximum concentration; CV, coefficient of variation; n, number; t½, terminal elimination half-life; T_max_, time to maximum concentration; Vz, volume of distribution. **Note:** Data in the table is for males and females combined.

**Table 4:** Plasma Pharmacokinetic Parameters Dose Normalized.

ALT-100 Dose Parameter (unit)	N	Mean (SD)	CV (%)
0.1 mg/kg (N=4)			
DNAUC_0-t_ (h*ug/mL/mg)	4	245.566 (490.1799)	199.6
DNC_max_ (ug/mL/mg)	4	0.6884 (0.6941)	100.8
0.4 mg/kg (N=4)			
DNAUC_0-t_ (h*ug/mL/mg)	4	88.935 (26.4757)	29.8
DNC_max_ (ug/mL/mg)	4	0.3769 (0.0594)	15.8
1.0 mg/kg (N=4)			
DNAUC_o-inf_ (h*ug/mL/mg)	1	154.944 (−)	-
DNAUC_0-t_ (h*ug/mL/mg)	6	118–833 (28.3668)	23.9
DNC_max_ (ug/mL/mg)	6	0.3924 (0.0885)	22.6
4.0 mg/kg (N=4)			
DNAUC_o-inf_ (h*ug/mL/mg)	5	122.198 (32.0502)	26.2
DNAUC_0-t_ (h*ug/mL/mg)	6	106.347 (30.4639)	28.6
DN C_max_ (ug/mL/mg)	6	0.3484 (0.0372)	10.7

**Abbreviations:** AUC0-t, area under the concentration-time curve from time zero (Predose) to last quantifiable concentration at time t; AUC0-inf, area under the concentration-time curve from time zero (Predose) extrapolated to infinity; CL, total body clearance; C_max_, maximum concentration; CV, coefficient of variation; n, number; t½, terminal elimination half-life; Tmax, time to maximum concentration; Vz, volume of distribution.

## Data Availability

The data that support the findings of this study are available from the corresponding author upon reasonable request. Data collected for the study, including deidentified participant data and a data dictionary defining each field in the set, will be made available to others upon formal request and with a signed material transfer agreement. Upon formal request and with a signed material transfer agreement related documents will be available (e.g., study protocol, statistical analysis plan, informed consent form). The data may be requested upon publication. The data will only be shared via individual secured network connections.
